# Radiomics nomogram combined with clinical factors for predicting pathological complete response in resectable esophageal squamous cell carcinoma

**DOI:** 10.3389/fonc.2024.1347650

**Published:** 2024-10-31

**Authors:** Zihao Lu, Yongsen Li, Wenxuan Hu, Yonghao Cao, Xin Lv, Xinyu Jia, Shiyu Shen, Jun Zhao, Chun Xu

**Affiliations:** Department of Thoracic Surgery, The First Affiliated Hospital of Soochow University, Suzhou, China

**Keywords:** radiomics, nomogram, pathological complete response, ESCC, neoadjuvant immunochemotherapy

## Abstract

**Introduction:**

Predicting the efficacy of neoadjuvant immunochemotherapy (NICT) for esophageal squamous cell carcinoma (ESSC) prior to surgery can minimize unnecessary surgical interventions and facilitate personalized treatment strategies. Our goal is to develop and validate an image-based radiomic model using preoperative computed tomography (CT) scans and clinical data to predict pathological complete response (pCR) in resectable ESSC following neoadjuvant immunotherapy.

**Methods:**

We retrospectively collected data from patients diagnosed with ESCC at the First Affiliated Hospital of Soochow University between January 2018 and May 2023, who received preoperative neoadjuvant immunochemotherapy. Eligible patients were randomly divided into training and validation sets. Radiomic features extracted from preprocessed CT images were used to develop a radiomic model, incorporating Radiomic score (Rad-score) and clinical factors through multivariate logistic regression analysis. The model’s performance was assessed for calibration, discrimination, and clinical utility in an independent validation cohort.

**Results:**

We enrolled a total of 105 eligible participants who were randomly divided into two groups: a training set (N=74) and a validation set (N=31). After data dimension reduction and feature selection, we identified 11 radiomic features, which collectively formed the Rad-score. Rad-score had an area under the curve (AUC) of 0.83 (95% CI 0.72-0.93) in the training set and 0.78 (95% CI 0.60-0.95) in the validation set. Multivariate analysis revealed that radiological response and Neutrophil–Lymphocyte Ratio (NLR) were independent predictors of pCR, with p-values of 0.0026 and 0.0414, respectively. We developed and validated a nomogram combining Rad-score and clinical features, achieving AUCs of 0.90 (95% CI 0.82-0.98) in the training set and 0.85 (95% CI 0.70-0.99) in the validation set. The Delong test confirmed the nomogram’s superiority over pure radiomic and clinical models. Decision curve analysis (DCA) and integrated discrimination improvement (IDI) assessment supported the clinical value and superiority of the combined model.

**Conclusion:**

The nomogram, which integrates Rad-score and clinical features, offers a precise and reliable method for predicting pCR status in ESCC patients who have undergone neoadjuvant immunochemotherapy. This tool aids in tailoring treatment strategies to individual patients.

## 1 Introduction

Esophageal cancer (EC) has emerged as the seventh most prevalent cancer globally and ranks sixth among the leading causes of cancer-related mortality (1). China is responsible for over half of the annual new cases of EC worldwide, with more than 90% of EC cases being diagnosed as esophageal squamous cell carcinoma (ESCC). The lack of early clinical symptoms often leads to advanced stage diagnosis in many ESCC patients (2). Although surgery remains the main treatment for resectable EC, recent studies conduct both domestically and internationally has demonstrated significant advantages in combining neoadjuvant therapy with surgery for these patients (3, 4). The emergence of immunotherapy has ushered in a new era in the treatment of advanced EC, with immune checkpoint inhibitors (ICIs) showing promising efficacy (5–7). The utilization of neoadjuvant immunotherapy in EC treatment is garnering heightened interest, as early studies have indicated that combining neoadjuvant immunotherapy with chemotherapy is both safe and feasible for EC patients. These studies have also shown promising recent results, suggesting a positive prognosis (8, 9). Treatment responses in EC patients are highly individualized, with achieving a pathological complete response (pCR) being a crucial predictor of positive outcomes (10–12). Despite promising results from neoadjuvant immunotherapy combined with chemotherapy, only approximately one-third of patients achieve pCR (8, 9). Esophagectomy, a procedure commonly used in treatment, is associated with a high rate of complications, including a postoperative complication rate (Clavien-Dindo grade ≥3) of up to 60% and a mortality rate as high as 5%, which can greatly impact patients’ quality of life (13, 14). A meta-analysis has demonstrated that surgical intervention following clinical complete remission from neoadjuvant therapy for EC does not confer long-term survival advantages over non-surgical treatments (15). Recent research has shifted towards organ preservation strategies, suggesting that for patients achieving pCR, active surveillance and organ preservation techniques may serve as viable alternatives.

Traditional imaging modalities have limitations in accurately predicting pCR in EC following neoadjuvant therapy (16). The Response Evaluation Criteria in Solid Tumors (RECIST) scoring system is frequently employed to assess treatment response in solid tumors, but discrepancies exist between RECIST scores and pathological outcomes (17). Research has indicated that blood biomarkers such as the nNLR, may offer a more reliable means of predicting the pathological response to neoadjuvant therapy (18, 19). However, the reliability of these serum biomarkers is often compromised by various factors such as inflammation or infection, highlighting the necessity for more robust biomarkers to assess the efficacy of neoadjuvant immunochemotherapy in EC, particularly those that can be identified through non-invasive methods.

The rapid progression of medical imaging technologies, data algorithms, and analytics has facilitated the possibility of large-scale data mining and analysis of medical images. The concept of radiomics, initially proposed by Lambin, allows for the high-throughput extraction of numerous quantitative image features from medical images, providing valuable **Supplementary Information** for purposes such as disease diagnosis, prognostic analysis, and treatment response prediction (20–22). Several studies have utilized radiomics to forecast responses to neoadjuvant therapy in various types of cancers, resulting in promising outcomes (23–25). Prior studies have not examined the potential value of radiomics in predicting pCR after neoadjuvant immunochemotherapy in ESCC. Therefore, our objective was to develop a composite model utilizing radiomic features extracted from pre-treatment CT scans along with clinical factors to predict pCR after neoadjuvant immunochemotherapy in ESCC. This model accurately identifies suitable subgroups within the clinical population, ensuring localized control and the potential avoidance of surgery in specific patients, thereby maintaining organ integrity and ultimately improving overall quality of life for these individuals.

## 2 Materials and methods

**Figure 1:**
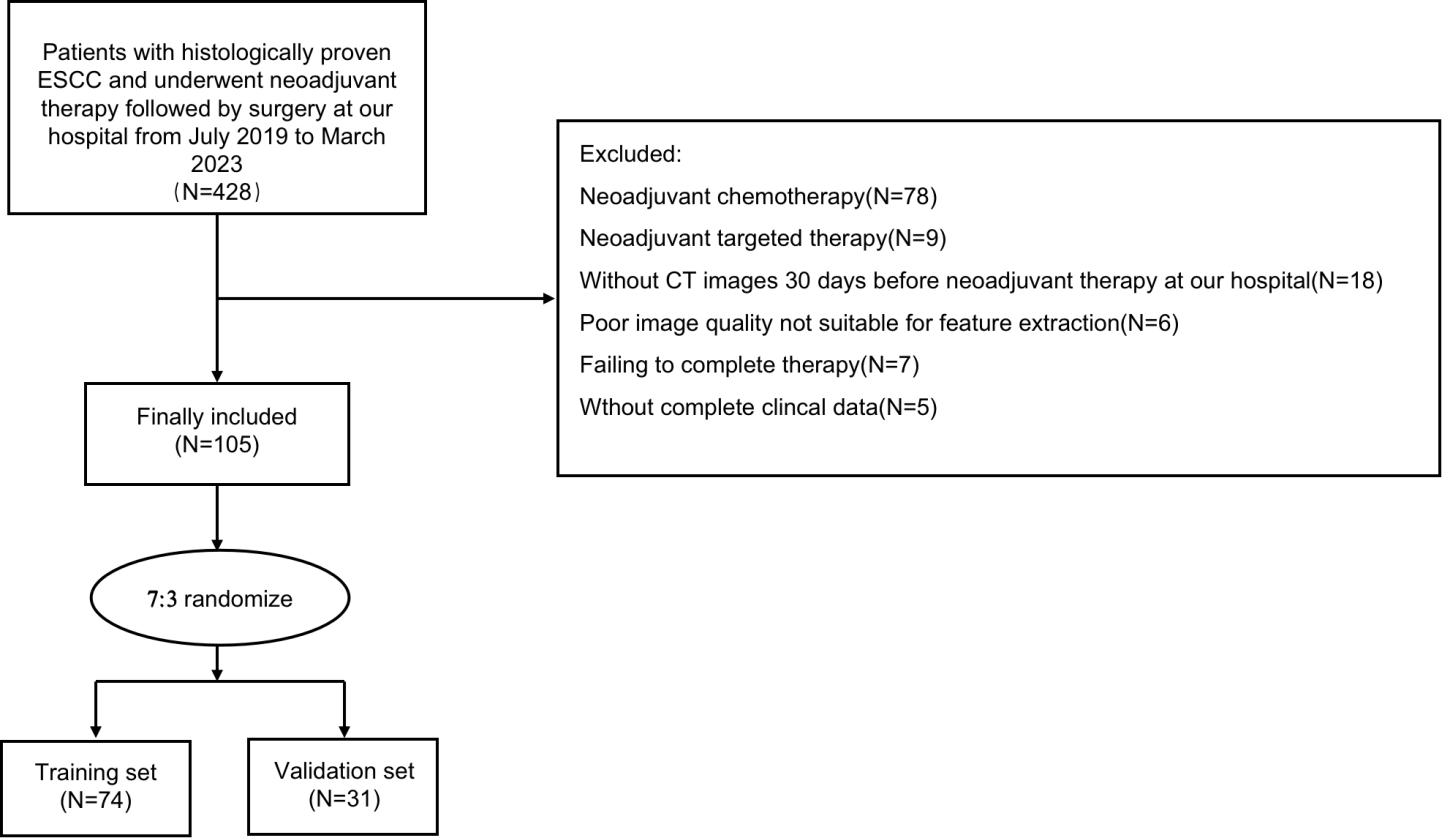
Patient selection and distribution flowchart.

**Figure 2:**
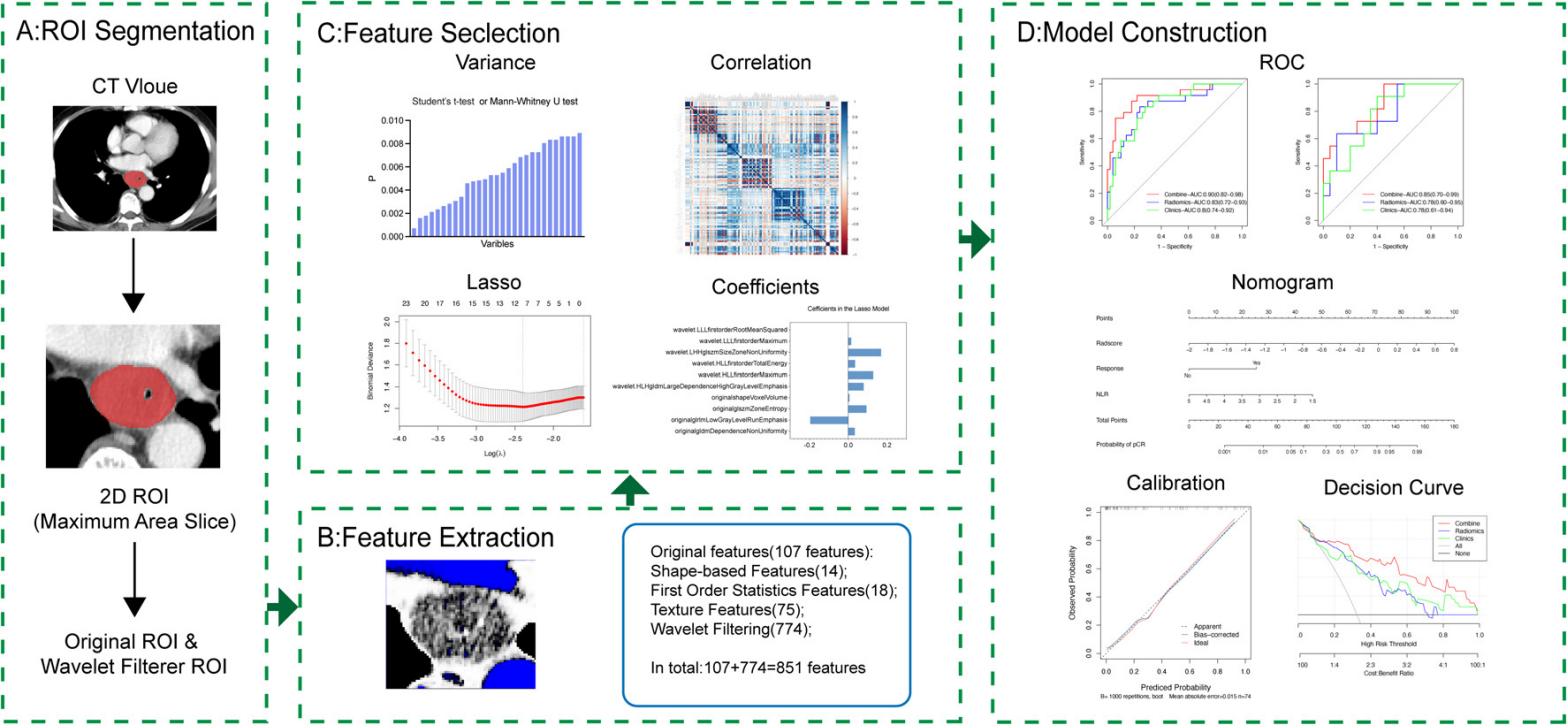
Workflow of radiomics analysis.

The patient selection and distribution process were illustrated in **Figure 1**, and the radiomics analysis process was depicted in **Figure 2**.

### 2.1 Patient

Patients with a diagnosis of with ESCC who received neoadjuvant immunotherapy combined with chemotherapy at the First Affiliated Hospital of Soochow University between January 2018 and May 2023 were retrospectively included in this study. Inclusion criteria for our study were as follows: (1) Age 18 years or older, (2) Undergoing surgery after neoadjuvant immunotherapy, (3) Clinical staging of II - IV A, (4) Preoperative histological confirmation of ESCC, (5) Scored within the range of 0 to 2 according to the ECOG performance status criteria. Patients meeting any of the following exclusion criteria were excluded: (1) Preoperative pathology confirming adenocarcinoma, (2) Incomplete treatment, including patients who refused surgery or temporarily halted treatment due to immunotherapy-related adverse effects, (3) Preoperative identification of distant metastases, (4) Incomplete clinical information, (5) Lack of pre- and post-treatment CT image data or poor CT image quality, and (6) Concurrent malignancies. Ultimately, a total of 105 patients were enrolled, with participants randomly allocated into training (N=74) and validation (N=31) sets at a ratio of 7:3.

### 2.2 Treatment groups

All patients underwent thorough pretreatment evaluation, including physical examinations, standard laboratory tests, pulmonary function assessments, Endoscopic Gastrointestinal Decontamination (EGD) with endoscopic ultrasound and biopsy, and enhanced chest/abdominal CT and PET scans (if available) for staging according to the 8th edition of AJCC TNM classification. A multidisciplinary team assessed patient suitability for surgery after neoadjuvant therapy. In this study, all patients underwent a two-cycle regimen of platinum-based chemotherapy combined with taxanes (docetaxel or paclitaxel), concurrently administered with 200mg of sintilimab/tislelizumab. All patients received the complete combination therapy of immunotherapy and chemotherapy. All patients underwent a CT examination within one week before surgery to assess tumor reduction rate, following the criteria of RECIST 1.1. Response was evaluated by the relative change in baseline and post-treatment diameters using CT, with a reduction of 30% or greater considered indicative of a positive response and all other cases categorized as non-responsive. After completing treatment, patients underwent Ivor-Lewis esophagectomy with lymph node dissection and gastric conduit reconstruction. Pathological response to neoadjuvant immunochemotherapy was assessed by two independent pathologists, with pCR defined as the absence of viable cancer cells.

### 2.3 CT data acquisition and retrieval procedure

All patients underwent contrast-enhanced CT (CE-CT) scans at two time points: one week prior to the initiation of neoadjuvant Immuno-Chemotherapy and one week prior to surgery, referred as pre-treatment and preoperative CE-CT scans, respectively. Imaging was conducted using a GE Lightspeed 64-slice spiral CT scanner (GE Healthcare, Milwaukee, Wisconsin) with specific parameters: tube voltage of 120 kV, tube current of 120 mAs, gantry rotation time of 0.6 seconds, detector collimation of 64×0.625 mm, field of view ranging from 400 to 500 mm, matrix size of 512×512, slice thickness of 5 mm, and interslice gap of 5 mm. A contrast agent was administered intravenously at a rate of at 3.0 ml/s (dose of 1-1.5 ml/kg, utilizing iopromide injection at a concentration of 300 mg iodine/ml) using a high-pressure injector. This was followed by a saline flush of 30-40 ml, and late arterial phase CT images were obtained after a delay of 30 seconds.

### 2.4 Tumor masking and radiomic feature extraction

In order to define regions of interest (ROIs) for subsequent radiomic analysis, post-contrast CT images were manually segmented using the open-source software, ITK-SNAP (http://www.itksnap.org/pmwiki/pmwiki.php). The 2D ROIs were delineated on the slice with the maximum tumor axis diameter and then imported into 3D Slicer (https://www.slicer.org/) for further analysis. To minimize inter-image variability, scans were resampled to a voxel size of 1×1 millimeter prior to feature extraction.

A total of 851 radiomic features were extracted, comprising 162 first-order statistics, 14 shape-based features, 216 gray-level co-occurrence matrix (GLCM) features, 144 gray-level run length matrix (GLRLM) features, 144 gray-level size zone matrix (GLSZM) features, 126 gray-level dependence matrix (GLDM) features, and 45 neighborhood gray-tone difference matrix (NGTDM) features.

To assess the reliability of radiomic features, tumor segmentation was independently performed by a radiologic oncologist and another radiologist. Reproducibility was assessed by having the same observer repeat tumor segmentation for 30 randomly chosen patients after a two-month interval, with consistency being evaluated using Intra-class correlation (ICC).

### 2.5 Feature selection

After features extraction, all the radiomic features were normalized using Z-score normalization by which the feature values were centered by removing the mean value of each feature, then divided by the standard deviation of each feature. The pre-processing made feature values lie within similar ranges, which reduced the influence of features with large discrete values. To reduce redundancy, we employed a three-step dimensionality reduction approach: (1) Univariate feature selection with T-tests or Mann-Whitney U-tests, retaining features with p-value < 0.05 related to pCR. (2) The intra-class correlation coefficient (ICC) analysis was performed to evaluate the reproducibility of each radiomics feature through Pearson and Spearman tests. Only the features with ICCs value ≥ 0.70 were selected for further analysis. (3) Regularized multivariate logistic regression with the least absolute shrinkage and selection operator (LASSO) was employed to select the best predictive features for pCR in the training set. With a linear combination of the selected features weighted by their respective coefficients, a model was used to estimate the immunochemotherapy outcomes based on the radiomic features. The model was defined as follows:


$$\text{y}=\sum_{\text{j}=1}^{\text{d}}{\text{β}}_{\text{j}}{\text{x}}_{\text{j}}+{\text{β}}_{0}+\text{ϵ}$$

Where y is 1 for patients with pCR and 0 for non-pCR patients; d is the number of features used in the model; ${\text{x}}_{\text{j}}\left(\text{j}=1,2,\ldots ,\text{d}\right)$ is the feature; ${\text{β}}_{\text{j}}\left(\text{j}=0,1,2,\ldots ,\text{d}\right)$ is the model parameter, and ϵ is the error term.

Using regularized regression to estimate the parameters of the model, feature selection (by forcing many parameters to zero value) can be performed simultaneously. The aim of this approach is to minimize the cost function:


$$\sum_{i=1}^{N}{\left[y_{i}-S\left(\sum_{j=1}^{d}\beta_{j}x_{ij}+\beta_{0}\right)\right]}^{2}+\lambda \sum_{j=1}^{d}\left|\left|\beta_{j}\right|\right|$$

Where ${\text{y}}_{\text{i}}$ is the outcome of patient i, N is the number of patients, S is the sigmoid function, ${\text{x}}_{\text{ij}}$ is the jth feature of the ith patient, and λ is the regularization parameter which chosen via five-fold cross-validation, minimizing mean squared error. The sigmoid function S is defined as follows:


$$\text{S}\left(\text{x}\right)=\frac{1}{1+{\text{e}}^{-\text{x}}}$$

with the LASSO penalty $\sum_{\text{j}=1}^{\text{d}}\left|\left|{\text{β}}_{\text{j}}\right|\right|$ applied, leading to sparse models by setting some parameters ( ${\text{β}}_{\text{j}}\text{s}$ ) to zero. Features with greater contributions to the model are selected. Subsequently, we calculated the Radiomic score (Rad-score) for each patient based on the weighted coefficients from the LASSO regression model in the training set. The LASSO logistic regression formula:


$$\text{Rad-score}=\beta_{0}+\beta_{1}x_{1}+\beta_{2}x_{2}+\beta_{3}x_{3}+\cdots +\beta_{n}x_{n}$$

In the above formula, x_n_ represents the radiomics feature identified by the LASSO logistic regression model, β_0_ is the constant for Rad-score, and β_n_ is the regression coefficient of the corresponding feature in the regression model. The Rad-score for each patient can be calculated according to the formula. Receiver operating characteristic (ROC) curve analysis was used to assess the performance of Rad-score.

### 2.6 Model construction and validation

Within the training set, radiomic, clinical, and combined radiomic-clinical models were developed to predict pCR. Optimal clinical variables for pCR prediction were identified through a combination of univariate and multivariate logistic regression analysis, encompassing factors such as age, gender, BMI, tumor characteristics, smoking, alcohol consumption, cancer staging, neoadjuvant immunochemotherapy details, and blood markers. Model performance was evaluated using AUC metric, with ROC curves comparisons conducted via the DeLong test. Superiority of the models was evaluated with integrated IDI and NRI, while model adequacy was tested with the Hosmer-Lemeshow test. Clinical usefulness was assessed with Decision curve analysis (DCA).

### 2.7 Statistical analysis

Statistical analysis was carried out using IBM SPSS version 20.0 and R software version 4.1.3. For continuous variables, we used t-tests or Mann-Whitney U tests, while chi-square tests or Fisher’s exact tests were used for categorical variables. Clinical variables predicting pCR were determined through univariate and multivariate logistic regression, including Lasso algorithm for variable selection. Model development, nomograms, and calibration plots were created using the ‘rms’ package. Internal validation was performed, and ROC comparisons were done using the ‘pROC’ package. DCA used the ‘rmda’ package, and IDI and NRI were computed with the ‘PredictABEL’ package. Statistical significance was set at p-value < 0.05.

## 3 Results

### 3.1 Baseline characteristics

A total of 105 patients were enrolled in this study, comprising 74 in the training set and 31 in the validation set (**Table 1**). The age in all patients was 66.99 (6.11) years, with 84 (80.0%) males and 21 (20.0%) females. The majority of patients were in stage III (64 cases, 61.0%), and 61 cases (58.1%) exhibited radiological evidence of response. The pCR rates in the training and validation sets were 32.4% and 35.5%, respectively. There were no significant differences in clinical characteristics between the training and validation sets (**Supplementary Table 1**), confirming their comparability for use as training and validation datasets.

**Table 1:** Clinicopathological characteristics of the dataset.

Characteristic	Patients, No. (%)						
	Institution 1 (training set)			Institution 2 (validation set)			All patients
	Non-pCR, N = 50	pCR, N = 24	P-value^1^	Non-pCR, N = 20	pCR, N = 11	P-value^1^	N = 105
Age, Mean (SD)	67.58 (5.65)	66.46 (5.76)	0.57	66.35 (7.18)	66.64 (7.39)	0.90	66.99 (6.11)
Sex, n (%)			0.54			0.38	
Female	9 (18%)	6 (25%)		5 (25%)	1 (9.1%)		21 (20%)
Male	41 (82%)	18 (75%)		15 (75%)	10 (91%)		84 (80%)
BMI, Mean (SD)	22.41 (3.00)	22.95 (2.57)	0.27	23.05 (3.26)	22.94 (2.76)	0.73	22.71 (2.91)
Alcohol use, n (%)			0.93			0.42	
No	38 (76%)	18 (75%)		15 (75%)	6 (55%)		77 (73%)
Yes	12 (24%)	6 (25%)		5 (25%)	5 (45%)		28 (27%)
Tobacco use, n (%)			0.41			0.15	
No	34 (68%)	14 (58%)		13 (65%)	4 (36%)		65 (62%)
Yes	16 (32%)	10 (42%)		7 (35%)	7 (64%)		40 (38%)
Clinical T stagea, n (%)			0.15			>0.99	
2	16 (32%)	6 (25%)		4 (20%)	2 (18%)		28 (27%)
3	30 (60%)	12 (50%)		14 (70%)	8 (73%)		64 (61%)
4a	4 (8.0%)	6 (25%)		2 (10%)	1 (9.1%)		13 (12%)
Clinical N stagea, n (%)			0.28			0.54	
0	19 (38%)	5 (21%)		5 (25%)	3 (27%)		32 (30%)
1	24 (48%)	12 (50%)		9 (45%)	7 (64%)		52 (50%)
2	6 (12%)	6 (25%)		6 (30%)	1 (9.1%)		19 (18%)
3	1 (2.0%)	1 (4.2%)		0 (0%)	0 (0%)		2 (1.9%)
Clinical stage group, n (%)			0.18			0.87	
II	23 (46%)	10 (42%)		6 (30%)	3 (27%)		42 (40%)
III	21 (42%)	7 (29%)		12 (60%)	6 (55%)		46 (44%)
IV A	6 (12%)	7 (29%)		2 (10%)	2 (18%)		17 (16%)
Tumor location, n (%)			0.78			>0.99	
Proximal third	6 (12%)	4 (17%)		3 (15%)	1 (9.1%)		14 (13%)
Middle third	26 (52%)	13 (54%)		13 (65%)	8 (73%)		60 (57%)
Distal third	18 (36%)	7 (29%)		4 (20%)	2 (18%)		31 (30%)
Diameter, Mean (SD)	3.01 (0.64)	3.36 (0.57)	0.031	3.03 (0.60)	2.95 (0.74)	0.77	3.09 (0.64)
Histologic grade, n (%)			0.14			0.24	
G1+G2	22 (44%)	15 (63%)		11 (55%)	9(82%)		57 (54%)
G3	28 (56%)	9 (38%)		9 (45%)	2 (18%)		48 (46%)
Immunotherapy_Regimen, n (%)			0.14			0.81	
Sintilimab	32 (64%)	11 (50%)		10(40%)	6 (55%)		59 (56%)
Tisleizumab	18 (63%)	13 (50%)		10(35%)	5 (45%)		64 (44%)
Response, n (%)			<0.001			0.13	
No	30 (60%)	2 (8.3%)		10 (50%)	2 (18%)		44 (42%)
Yes	20 (40%)	22 (92%)		10 (50%)	9 (82%)		61 (58%)
NLR, Mean (SD)	3.03 (0.77)	2.57 (0.64)	0.018	2.88 (0.81)	2.30 (0.91)	0.066	2.82 (0.80)
PLR, Mean (SD)	154.33 (60.30)	162.06 (61.56)	0.27	140.22 (49.72)	131.95 (39.10)	0.73	151.07 (56.96)
LMR, Mean (SD)	3.58 (1.48)	3.18 (1.57)	0.39	3.53 (1.43)	3.97 (1.95)	0.74	3.52 (1.54)
PNI, Mean (SD)	46.55 (5.93)	46.50(4.85)	0.84	45.18 (5.45)	48.25 (2.83)	0.094	46.46 (5.35)
SCCA, Mean (SD)	1.43 (0.77)	1.53 (0.94)	0.88	2.03 (1.20)	1.57 (1.01)	0.25	1.58 (0.94)

^1^Wilcoxon rank sum test; Fisher’s exact test; Pearson’s Chi-squared test.

### 3.2 Model construction

#### 3.2.1 Radiomics feature selection and Rad-score construction

**Figure 3:**
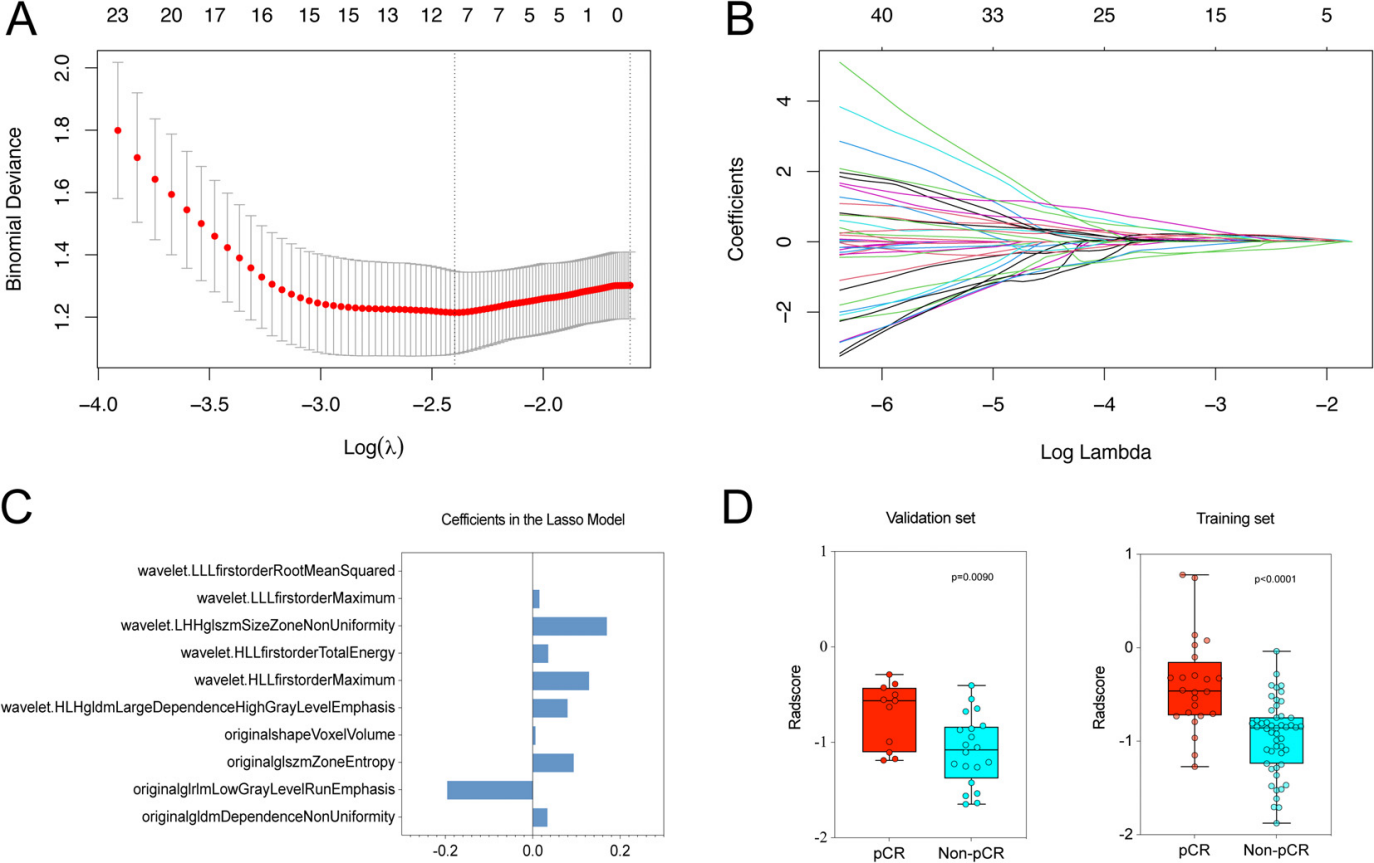
We utilized LASSO logistic regression to choose radiomic features for predicting pCR. **(A)** Optimal λ (lambda) value, selected through 5-fold cross-validation, was determined to be 0.091 at log(λ) = -2.398. **(B)** LASSO coefficient profile plot for radiomic features. **(C)** Final list of selected radiomic feature names and coefficients. **(D)** Rad-scores showed significant differences between pCR and Non-pCR groups in both the training (P < 0.001) and validation sets (P = 0.009).

Following an initial analysis of 851 radiomic features through differential and correlation analyses, a reduction to 98 features was achieved. Subsequent dimensionality reduction was carried out using the LASSO algorithm, resulting in the selection of optimal radiomic features for predicting pCR within the training set (**Figures 3A, B**). Ultimately, a total of 11 radiomic features were chosen with detailed information on feature names and coefficients provided in **Figure 3C**. The intra-observer and inter-observer ICC were calculated for these 11 features, all of which surpassed 0.800, suggesting their reliability and appropriateness for additional analysis (ICC range: 0.818–0.998, **Supplementary Table 2**). Following this, Rad-scores were determined for each patient. Significant differences in Rad-scores between patients with pCR and those without (Non-pCR) were observed in both the training (p < 0.001) and validation cohorts (P = 0.009) (**Figure 3D**).

#### 3.2.2 Model building

Univariate and multivariate logistic analyses (**Table 2**) were performed to determine clinical predictors of pCR status in the training set. Significant associations were observed between pCR status and tumor diameter, radiological response, and NLR in the univariate analysis. However, no correlations were found between pCR status and age, gender, tumor location, staging, platelet to lymphocyte ratio (PLR), prognostic nutritional index (PNI), or lymphocyte to monocyte ratio (LMR) (as indicated in **Table 2**). Multivariate analysis validated the influence of radiological response and NLR levels as independent factors on achieving pCR. Three distinct models were developed based on selected Rad-scores, clinical indicators, and their various combinations (radiomic only, clinical only, and radiomic-clinical combined models).

**Table 2:** Univariate and multivariate analysis of clinical data.

Variable	Univariate analysis		Multivariate analysis	
	OR (95%CI)	P-value	OR (95%CI)	P-value
Age	0.97(0.88-1.05)	0.424		
Sex	0.66(0.21-2.22)	0.49		
BMI	1.07(0.90-1.27)	0.45		
Alcohol use	1.06(0.32-3.20)	0.93		
Tobacco use	1.52 (0.55- 4.16)	0.42		
Clinical T stage				
2				
3	1.07(0.34- 3.55)	0.91		
4a	4.000.86-21.05)	0.08		
Clinical N stage				
0				
1	1.90(0.59-6.84)	0.30		
2	3.80(0.86-18.14)	0.08		
3	3.8(0.13-108.47)	0.37		
Clinical stage group				
II				
III	0.77(0.24-2.36)	0.65		
IV A	2.68(0.72-10.44)	0.14		
Tumor location				
Proximal third				
Middle third	0.75(0.18-3.37)	0.69		
Distal third	0.58(0.12- 2.86)	0.49		
Diameter	2.59(1.13-6.66)	0.03 *	1.83(0.63-5.76)	0.28
Histologic grade	0.47(0.17-1.26)	0.14		
G1+G2				
G3				
Immunotherapy_Regimen	0.74(0.78-5.75)	0.14		
Sintilimab				
Tisleizumab				
Response	16.50(4.23-110.33)	0.00041 ***	12.25(2.88-86.49)	0.0026 **
NLR	0.38(0.14-0.91)	0.04 *	0.38(0.14- 0.91)	0.041 *
PLR	1.00(0.99-1.01)	0.61		
LMR	0.83(0.58-1.16)	0.296		
PNI	1.00(0.91- 1.09)	0.970		
SCCA	1.15(0.63-2.08)	0.64		

*p<0.05 **p<0.01 ***p<0.001.

#### 3.2.3 The combined model demonstrates superior predictive capability

**Figure 4:**
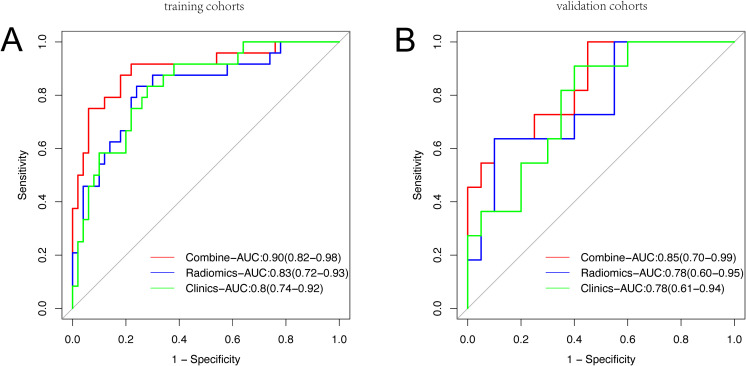
Receiver operating characteristic curve analysis of three models in the training set **(A)** and the validation set **(B)** for predicting Pathological Complete Response.

In this study, we conducted a comparison of ROC curves of three models within the training set (**Figure 4A**) and subsequently assessed their predictive performance for pCR in the validation set (**Figure 4B**). The AUC for the pure radiomic model in the training set was 0.83 (95% CI, 0.72-0.93), while the AUC for the pure clinical model was 0.80 (95% CI, 0.57-0.80). Following the training phase, both the radiomic-only and clinical-only models demonstrated accurate prediction of pCR in the validation set, achieving AUCs values of 0.78 (95% CI, 0.60-0.95) and 0.78 (95% CI, 0.61-0.94), respectively. The radiomic-clinical combined model exhibited superior performance, with AUCs of 0.90 (95% CI, 0.82-0.98) in the training set and 0.85 (95% CI, 0.70-0.99) in the validation set (**Supplementary Table 3**). This improvement was further substantiated by the Net Reclassification Index (NRI) and IDI (**Supplementary Table 4**). In the training cohort, the combined model exhibited superior performance compared to both the clinical and radiomic models, with NRI improvements of 0.26 and 0.17, and IDI improvement of 0.17 and 0.17, respectively. In the validation cohort, the combined model continued to show improved performance over the clinical model, with NRI and IDI improvements of 0.14 and 0.13, while outperforming the radiomic model with NRI and IDI improvements of 0.28 and 0.11, respectively.

#### 3.2.4 Radiomics-based nomogram construction

**Figure 5:**
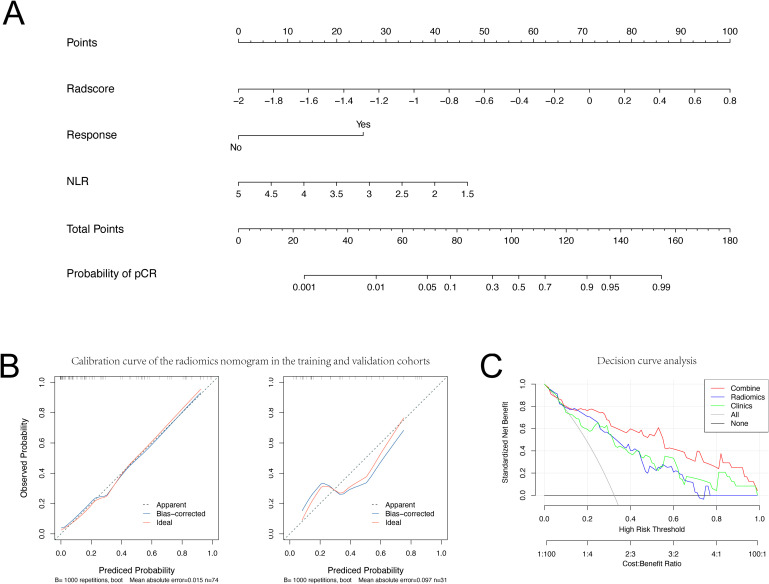
Nomogram, Calibration curve and Decision curve analysis. **(A)** Nomogram and **(B)** Calibration curve for Pathological Complete Response in the training and validation set. **(C)** Decision curve analysis for each model (clinical (Clinics) model, radiomics (Radiomics) model, and integrated (combined) model).

A radiomic-clinical combined model, incorporating Rad-scores and clinical data, and was illustrated as a nomogram (**Figure 5A**) to predict pCR following neoadjuvant immunochemotherapy.

#### 3.2.5 Performance and clinical utility of the constructed nomogram

The calibration curves of the pCR probability nomogram demonstrate strong agreement between predicted and observed values in both the training and validation cohorts (**Figure 5B**). The Hosmer-Lemeshow test, conducted with calibration curves, resulted in p-values greater than 0.05 for both the training and validation sets (P=0.210 and P=0.213, respectively), indicating a high level of fit between the model and the data.

Decision curves were utilized to assess the impact of the nomogram model on clinical treatment decisions. Analysis of the decision curves revealed that the nomogram model yielded greater benefit within a risk threshold range of 0.3 to 0.8, in contrast to the ‘treat-all’ or ‘no treatment’ strategies. Conversely, both the clinical and radiomic models exhibited lower net benefits than the nomogram model (**Figure 5C**).

## 4 Discussion

In the context of neoadjuvant immunochemotherapy for patients with EC, the ability to predict pCR prior to surgery facilitates the identification of specific subgroups who may benefit from alternative treatment approaches, such as organ-preserving strategies and active surveillance protocols. This tailored approach not only holds promise for sparing select patients from surgical resection but also aims to maintain long-term survival outcomes and improve overall quality of life. Following the completion of radiomic feature extraction, we selected the most effective features for predicting pCR using the LASSO algorithm. We then calculated the Radiomics score based on the weighted coefficients from the LASSO regression model, establishing a radiomics model. Furthermore, we identified two clinically relevant independent risk factors associated with pCR, namely NLR and Response, through univariate and multivariate logistic regression analyses, which contributed to the development of a clinical model. Moreover, we formulated a combined model that integrates clinical features and the Rad-score derived from radiomic features. The AUC values for the three models in the training dataset were 0.90, 0.83, and 0.80, respectively. Delong’s test revealed that the combined model exhibited significantly superior discriminative capacity compared to both the standalone radiomics and clinical models. Additionally, NRI and IDI showed substantial improvement in predictive performance for the combined model compared to both the standalone radiomics and clinical models. In the course of the study, a nomogram model incorporating clinical and radiological features, denoted as Rad-score, was developed and validated for its ability to forecast the pCR status of ESCC patients receiving neoadjuvant immunochemotherapy. The nomogram demonstrated strong predictive performance in both the training and validation cohorts, achieving AUC values of 0.90 and 0.85, respectively.

Tumors demonstrate variability in spatial and temporal characteristics (26, 27). Radiomics, as a non-invasive biomarker, has the capability to quantitatively analyze intratumoral heterogeneity (21). Previous studies indicate an association between pretreatment CT radiomic features and the efficacy of ICIs in solid tumor patients (28). Several current studies highlight the effectiveness of radiomic models in predicting the pathological response following neoadjuvant therapy in patients with EC (29, 30). The integration of immunotherapy with chemotherapy has been shown to synergistically enhance the immunogenicity of the tumor microenvironment (31). Multiple clinical studies have confirmed the safety and effectiveness of neoadjuvant immunochemotherapy in managing EC. This study represents the first attempt to predict pCR after neoadjuvant immunochemotherapy in potentially resectable ESCC using a radiomics-clinical combined method.

The preoperative assessment of the efficacy of neoadjuvant therapy is crucial for the subsequent development of individualized treatments. However, the presence of unconventional response patterns, such as pseudo-progression and hyper-progression, linked to immunotherapy, poses challenges to the accurate assessment of effectiveness using traditional response criteria. The dominant response criterion remains clinical complete response (cCR). Currently, there is no standardized diagnostic criteria for cCR, primarily denoting the absence of residual tumors confirmed through various non-surgical methods. Previous studies suggest that, for esophageal cancer (EC) patients achieving cCR after neoadjuvant therapy, the addition of esophagectomy can reduce short-term local recurrence rates, although no significant long-term survival benefits have been observed (32, 33). Pathological examination remains the gold standard for assessing treatment response. Despite employing various diagnostic methods for a comprehensive evaluation of cCR, the results still exhibit some inconsistency with pCR outcomes (34). Only one-third of cCR patients ultimately achieve pCR, potentially contributing to the higher local recurrence in non-surgical patients. For patients with EC, achieving pCR is associated with improved outcomes, indicating potentially better survival and lower local recurrence (35). The nomogram developed in this study incorporates Rad-score and two clinical parameters, which can be obtained through routine CT scans and blood tests. This model serves to assess the efficacy of neoadjuvant immunochemotherapy in EC patients, with higher nomogram scores suggesting a likelihood of achieving pCR. These patients may potentially avoid surgery without compromising long-term survival benefits, leading to an enhanced quality of life for the entire organ system.

Our research indicates that pre-treatment peripheral blood NLR is an independent prognostic indicator for pCR in ESCC patients following neoadjuvant immunotherapy. Increasing evidence supports the crucial role of tumor-associated inflammation in the host’s immune response to malignant tumors (36). The ratio of NLR, defined by the absolute counts of neutrophils and lymphocytes, may represent a balance between pro-tumor inflammatory status and anti-tumor immune response. ICristina et al. conducted a retrospective analysis to explore the association between pre-treatment peripheral blood NLR and response rates in patients undergoing ICI therapy. Their comprehensive pan-cancer analysis revealed that patients with elevated NLR levels experienced diminished efficacy of ICIs. Further validation in an independent cohort demonstrated a correlation between high NLR and lower rates of remission (17% vs. 28%) and clinical benefit (26% vs. 41%) (37). These findings may be associated with the tumor microenvironment. The observed associations between peripheral blood NLR and outcomes following Immune Checkpoint Inhibitor (ICI) therapy may be explained by a potential correlation between circulating neutrophils and neutrophils present in the tumor microenvironment (38). First, neutrophils are known to produce immunosuppressive factors and angiogenic molecules such as reactive oxygen species, vascular endothelial growth factor (VEGF), and matrix metalloproteinase 9 (MMP-9), which can promote tumor growth. The infiltration of neutrophils into the tumor microenvironment may play a role in creating a favorable environment for tumor progression (38, 39). Neutrophils have been implicated in tumor initiation, progression, and early dissemination, while decreased levels of circulating lymphocytes are associated with reduced tumor-infiltrating lymphocytes and a compromised anti-tumor T-cell response (40, 41). Additionally, an increased abundance of myeloid cells, including neutrophils, can exacerbate T-cell suppression (41, 42). These combined factors contribute to the establishment of an immunosuppressive tumor microenvironment, potentially reducing the efficacy of immune checkpoint inhibitors (ICIs). Concurrently, recent research has demonstrated that neutrophils in the peripheral blood can enhance the efficacy of distant metastasis by engaging with circulating tumor cells and amplifying their metastatic characteristics through promoting cell cycle advancement and hastening metastasis establishment (43, 44).Our study revealed NLR levels in both the pCR subgroup training set and validation set were significantly lower compared to those in the Non-pCR subgroup (2.57 vs. 3.03, 2.30 vs. 2.88).

In our study, a significant correlation was observed between the reduction in the maximum tumor diameter and pCR. Multiple studies have reported a notable association between the reduction in tumor volume after neoadjuvant therapy and the occurrence of pCR (45, 46). However, volumetric calculations are labor-intensive and time-consuming. We assessed the reduction rate in the maximum tumor diameter, a parameter easily calculated by measuring the CT lesion diameter. In the NICE trial, the CT-measured reduction in the longest lesion diameter was moderately positively correlated with the pathological regression rate (r=0.600) (9). Moreover, a recent study has assessed the relationship between CT measurements and the pathological response of esophageal squamous cell carcinoma patients. Their findings indicate that both the reduction rates in the long and short diameters are associated with the tumor’s pathological response. The reduction rates in both the long and short diameters effectively predict the pathological response of the tumor, with respective areas under the curve of 0.761 and 0.781 (47).In our study, 58% of patients exhibited a reduction in tumor diameter compared to pre-treatment levels, and this group demonstrated a pCR rate over four times higher than patients with no reduction in tumor diameter.

It is widely acknowledged that PD-L1 expression stands as one of the most commonly used immune therapy biomarkers. However, our study did not prioritize its inclusion, as our focus was on incorporating markers easily assessable in clinical settings. The clinical utility if PD-L1 expression is limited due to the substantial amount of tumor tissue required for its determination and the absence of a standardized quantitative scoring system for immune cells or PD-L1 immunohistochemistry (48). A *post hoc* analysis of JUPITER-06 and a meta-analysis have revealed that PD-L1 expression may not serve as a reliable biomarker for predicting immunotherapy efficacy. Despite the correlation between high PD-L1 expression in tumors and better tumor responses, its sensitivity is insufficient, given that a considerable portion of patients with low PD-L1 expression also achieved improved tumor responses (49). Moreover, various molecular and genomic biomarkers demonstrate predictive efficacy for immune therapy in diverse cancer types. These include tumor mutational burden (TMB), specific gene mutations, human leukocyte antigen class I zygosity and diversity, and microsatellite instability (MSI) status (50, 51). However, these biomarkers have limitations that hinder their widespread clinical use, such as the need for sufficient tumor tissue and tumor DNA sequencing (52). There is a need for the development of predictive biomarkers that are easily accessible at low cost, applicable in diverse environments, and independent of advanced genomic technologies or specialized histopathologic expertise.

## 5 Limitation

This study has certain limitations. Firstly, this is a retrospective study and the possibility of selection bias during data collection cannot be ruled out. Therefore, a prospective study is needed for further validation. Secondly, the small sample size of pCR patients relative to the entire cohort, all from a single center, may pose challenges in balancing baseline characteristics when conducting randomization. Moreover, the majority of participants in the study were male individuals diagnosed with middle esophageal cancers. Our model may demonstrate improved applicability to this specific demographic, while further validation is necessary to assess its effectiveness in female patients with upper and lower esophageal cancers. Additionally, the potential impact of dynamic changes in radiomics (delta radiomics) and other relevant clinicopathological factors, including tumor infiltrating lymphocytes, gene mutation profiles, PET images, and serum densities of specific immune cell subgroups, remains unexplored. Furthermore, it is advisable to explore larger datasets from multiple centers to validate the robustness and reproducibility of the proposed radiomics model through multicenter studies and randomized controlled clinical trials. Lastly, the limited spatial resolution of CT, may introduce bias in determining the boundaries between lesions and normal esophageal tissue during manual ROI segmentation. Future efforts should explore and develop accurate automated segmentation methods.

## Data Availability

The raw data supporting the conclusions of this article will be made available by the authors, without undue reservation.
