# Supplementary material for: Radiomics nomogram combined with clinical factors for predicting pathological complete response in resectable esophageal squamous cell carcinoma
Source: Front Oncol. 2024 Oct 31;14:1347650. doi: 10.3389/fonc.2024.1347650 (PMC11560869; doi:10.3389/fonc.2024.1347650)
Supplement: Supplementary file 1 [file Table1.docx]

Supplementary Table 1

| **Characteristic** | Patients, No. (%) | | | |
| --- | --- | --- | --- | --- |
|  | **Overall(** N = 105) | **Training set(**N = 74) | **Validation set(**N = 31) | **P-value**^1^ |
| pCR, n (%) | 35 (33%) | 24 (32%) | 11 (35%) | 0.76 |
| Age, Mean (SD) | 66.99 (6.11) | 67.22 (5.67) | 66.45 (7.13) | 0.91 |
| Sex, n (%) |  |  |  | 0.91 |
| Female | 21 (20%) | 15 (20%) | 6 (19%) |  |
| Male | 84 (80%) | 59 (80%) | 25 (81%) |  |
| BMI, Mean (SD) | 22.71 (2.91) | 22.58 (2.86) | 23.01 (3.04) | 0.45 |
| Alcohol use, n (%) |  |  |  | 0.40 |
| No | 77 (73%) | 56 (76%) | 21 (68%) |  |
| Yes | 28 (27%) | 18 (24%) | 10 (32%) |  |
| Tobacco use, n (%) |  |  |  | 0.33 |
| No | 65 (62%) | 48 (65%) | 17 (55%) |  |
| Yes | 40 (38%) | 26 (35%) | 14 (45%) |  |
| Clinical T stagea, n (%) |  |  |  | 0.45 |
| 2 | 28 (27%) | 22 (30%) | 6 (19%) |  |
| 3 | 64 (61%) | 42 (57%) | 22 (71%) |  |
| 4a | 13 (12%) | 10 (14%) | 3 (9.7%) |  |
| Clinical N stagea, n (%) |  |  |  | 0.76 |
| 0 | 32 (30%) | 24 (32%) | 8 (26%) |  |
| 1 | 52 (50%) | 36 (49%) | 16 (52%) |  |
| 2 | 19 (18%) | 12 (16%) | 7 (23%) |  |
| 3 | 2 (1.9%) | 2 (2.7%) | 0 (0%) |  |
| Clinical stage group, n (%) |  |  |  | 0.16 |
| II | 42 (40%) | 33 (45%) | 9 (29%) |  |
| III | 46 (44%) | 28 (38%) | 18 (58%) |  |
| IV A | 17 (16%) | 13 (18%) | 4 (13%) |  |
| Tumor location, n (%) |  |  |  | 0.30 |
| Proximal third | 14 (13%) | 10 (14%) | 4 (13%) |  |
| Middle third | 60 (57%) | 39 (53%) | 21 (68%) |  |
| Distal third | 31 (30%) | 25 (34%) | 6 (19%) |  |
| length, Mean (SD) | 3.09 (0.64) | 3.13 (0.64) | 3.00 (0.64) | 0.34 |
| Histologic grade, n (%) |  |  |  | 0.17 |
| G1+G2 | 57 (54%) | 37 (50%) | 20 (65%) |  |
| G3 | 48 (46%) | 37 (50%) | 11 (35%) |  |
| Immunotherapy_Regimen, n (%) |  |  |  | 0.54 |
| Sintilimab | 59 (56%) | 43 (58%) | 16 (52%) |  |
| Tisleizumab | 46 (44%) | 31 (42%) | 15 (48%) |  |
| Resist, n (%) |  |  |  | 0.67 |
| No | 44 (42%) | 32 (43%) | 12 (39%) |  |
| Yes | 61 (58%) | 42 (57%) | 19 (61%) |  |
| NLRg, Mean (SD) | 2.82 (0.80) | 2.88 (0.76) | 2.68 (0.87) | 0.21 |
| PLR, Mean (SD) | 151.07 (56.96) | 156.84 (60.40) | 137.28 (45.73) | 0.056 |
| LMR, Mean (SD) | 3.52 (1.54) | 3.45 (1.51) | 3.69 (1.61) | 0.59 |
| PNI, Mean (SD) | 46.46 (5.35) | 46.54 (5.57) | 46.27 (4.87) | 0.97 |
| SCCA, Mean (SD) | 1.58 (0.94) | 1.46 (0.82) | 1.86 (1.14) | 0.080 |
| ^1^Pearson's Chi-squared test; Wilcoxon rank sum test; Fisher's exact test | | | | |
