# Supplementary material for: Radiomics nomogram combined with clinical factors for predicting pathological complete response in resectable esophageal squamous cell carcinoma
Source: Front Oncol. 2024 Oct 31;14:1347650. doi: 10.3389/fonc.2024.1347650 (PMC11560869; doi:10.3389/fonc.2024.1347650)
Supplement: Supplementary file 2 [file Table2.docx]

Supplementary Table 2

| Feature ICC | ICC-intra | ICC-inter |
| --- | --- | --- |
| originalgldmDependenceNonUniformity | 0.818 | 0.861 |
| originalglrlmLowGrayLevelRunEmphasis | 0.948 | 0.940 |
| originalglszmZoneEntropy | 0.909 | 0.851 |
| originalshapeVoxelVolume | 0.869 | 0.870 |
| wavelet.HLHgldmLargeDependenceHighGrayLevelEmphasis | 0.930 | 0.947 |
| wavelet.HLLfirstorderMaximum | 0.974 | 0.965 |
| wavelet.HLLfirstorderTotalEnergy | 0.918 | 0.927 |
| wavelet.LHHglszmSizeZoneNonUniformity | 0.961 | 0.948 |
| wavelet.LLLfirstorderMaximum | 0.998 | 0.996 |
| wavelet.LLLfirstorderRootMeanSquared | 0.994 | 0.988 |
