# Supplementary material for: Radiomics nomogram combined with clinical factors for predicting pathological complete response in resectable esophageal squamous cell carcinoma
Source: Front Oncol. 2024 Oct 31;14:1347650. doi: 10.3389/fonc.2024.1347650 (PMC11560869; doi:10.3389/fonc.2024.1347650)
Supplement: Supplementary file 3 [file Table3.docx]

Supplementary Table 3

| Models | Training set (N=74 ) | | | P | Validation set (N=31) | | | P |
| --- | --- | --- | --- | --- | --- | --- | --- | --- |
|  | AUC | (95%CI) | |  | AUC | (95%CI) | |  |
|  |  | Lower | Upper |  |  | Lower | Upper |  |
| Combined | 0.90 | 0.82 | 0.98 |  | 0.85 | 0.70 | 0.99 |  |
| Radiomics | 0.83 | 0.72 | 0.93 |  | 0.78 | 0.60 | 0.95 |  |
| Clinics | 0.80 | 0.74 | 0.93 |  | 0.78 | 0.61 | 0.94 |  |
| Combined versus Radiomics |  |  |  | 0.08 |  |  |  | 0.19 |
| Combined versus Clinics |  |  |  | 0.05 |  |  |  | 0.24 |
| Radiomics versus Clinics |  |  |  | 0.91 |  |  |  | 0.99 |
