# Supplementary material for: Radiomics nomogram combined with clinical factors for predicting pathological complete response in resectable esophageal squamous cell carcinoma
Source: Front Oncol. 2024 Oct 31;14:1347650. doi: 10.3389/fonc.2024.1347650 (PMC11560869; doi:10.3389/fonc.2024.1347650)
Supplement: Supplementary file 4 [file Table4.docx]

Supplementary Table 4

| Models | Training set | | Validation set | |
| --- | --- | --- | --- | --- |
|  | NRI | IDI | NRI | IDI |
| Combined versus Radiomics | 0.17 ( -0.06 - 0.39 ) | 0.17( 0.09 - 0.25) | 0.28( -0.15 - 0.72 ) | 0.11( 0.01 - 0.21 ) |
| Combined versus Clinics | 0.26 ( -0.007 - 0.53 ) | 0.17( 0.08 - 0.27 ) | 0.14 ( -0.26 - 0.54 ) | 0.13 ( 0.02 - 0.24 ) |
